# Dispersive Liquid–Liquid Microextraction Method Utilizing a Novel Peripherally Tetra-Substituted Ni(II) Phthalocyanine as a Sensor Prior to UV-Visible Spectrophotometry for the Determination of Co^2+^

**DOI:** 10.3390/molecules30122548

**Published:** 2025-06-11

**Authors:** Yasemin Çağlar, Ece Tuğba Saka

**Affiliations:** 1 Department of Genetic and Bioengineering, Giresun University, Giresun 28200, Türkiye; 2 Department of Chemistry, Karadeniz Technical University, Trabzon 61080, Türkiye; esaka@ktu.edu.tr

**Keywords:** DLLME, phthalocyanine, Co^2+^, anthracene

## Abstract

Dispersive liquid–liquid microextraction (DLLME) is an economical, rapid, sensitive, and environmentally friendly miniaturized liquid–liquid extraction format. It has been successfully applied in trace element analysis since 2006 when it was first proposed. This article describes a new dispersive liquid–liquid microextraction method for the determination of trace amounts of Co^2+^. In brief, this method involves the extraction of Co^2+^ from the sample to the trichloromethane phase by the dispersive action of methanol after the formation of a complex with a novel 9-(methylaminomethyl)anthracene-Ni(II) phthalocyanine (MAMA Ni(II)Pc **2**) as a sensor. The first step in this study was the synthesis and characterisation of the sensor. Later, the proposed method was optimized with respect to various parameters such as extraction and dispersive solvents and their amounts, pH, sensor concentration, and centrifugation time and rate. The calibration graph was linear between 0.40 and 260 µg/L, with an R^2^ of 0.9978. The limit of detection and limit of quantification were found to be 0.19 µg/L and 0.46 µg/L, respectively. To evaluate the precision of this method, the analysis of a 50 µg/L Co^2+^ solution was carried out. The intra-day and inter-day relative standard deviation values were calculated as 1.7% and 2.4%, respectively (n = 7). The accuracy of the proposed method was investigated by means of a standard addition/recovery test.

## 1. Introduction

The close relationship between trace metal levels and living organisms has created a need to determine trace element levels in environmental samples. Due to the variety and complexity of environmental samples, it is very difficult to determine the methodology and techniques to be used for trace element analysis [1,2]. Cobalt is an essential trace element for humans and can be of both natural and anthropogenic origin. Volcanic eruptions, mining, forest fires, metallic layers on the seabed, rocks, and plants are the main natural sources of cobalt [3]. The principal anthropogenic sources that increase the concentration of cobalt in the environment are the production of rechargeable batteries, catalysts, pharmaceuticals, and corrosion-resistant alloys [4,5].

In the human body, cobalt is found in some enzymes and is a consequential component of vitamin B12 (cobalamin). It also boosts immunity and has antibacterial activity [6,7]. Cobalt deficiency in the human system can lead to several different types of anemia. On the other hand, high doses of cobalt may give rise to symptoms such as thyroid disorders, cardiovascular complaints, diarrhea, inhibition of enzyme activity, irritation of the gastrointestinal tract, and nausea [8,9,10]. The amount of cobalt to be ingested daily is given as 5–50 μg/day. As with any other trace element, even small amounts of cobalt can accumulate in the organs and cause unfavourable health effects over time [11,12]. Human exposure to cobalt is closely linked to the daily diet. It is vital to construct an accurate, sensitive, hasty, and eco-friendly procedure for the evaluation of cobalt levels in different food matrices [13]. An extensive array of analytical approaches has been proposed for the determination of trace amounts of cobalt. However, each of these techniques has certain limitations. Inductively coupled plasma mass spectrometry (ICP-MS) [14,15] and inductively coupled plasma optical emission spectroscopy (ICP-OES) [16,17,18] are constrained by spectral interferences and high operational costs. Atomic absorption spectrometry (AAS) [19,20] and electrothermal atomic absorption spectrometry (ET-AAS) [21] require a certain level of expertise. In comparison with the aforementioned techniques, UV-Vis spectroscopy [22,23] can be considered a favourable alternative, as it is a low-cost, rapid, and environmentally friendly method that can be performed with basic-level expertise.

To be able to measure trace amounts of metal ions in complex matrices in a precise and accurate way, pre-treatment is often necessary. More recently, miniaturised liquid phase extraction techniques, which are fast, inexpensive, have a high enrichment factor, and allow for the use of small volumes of solvent, have offered very suitable alternatives for pre-treatment [24,25,26,27]. The DLLME approach has been widely used for the determination of trace analytes in aqueous samples since 2006 due to its simplicity, high extraction capacity, use of organic solvents in micro-volumes, and relatively low cost. In the DLLME method, grounded in the ternary solvent system, firstly, a suitable mixture of extraction and dispersive solvents is quickly transferred into the watery sample solution with the help of an injector. This generates a turbid solution, facilitating swift extraction considering the significant surface area interaction between the extractive and aqueous phases. The small droplets of extraction solvent containing the analyte are collected at the bottom of the test tube via centrifugation. Finally, the analyte is quantitatively determined using the appropriate instrumental technique [28,29,30,31,32].

Phthalocyanines (Pc) are macrocyclic molecules consisting of four iminoisoindole units linked by aza-bridges. Their delocalized 18-π electron structure gives them striking and adjustable optical characteristics, such as strong absorption of light in the red part of the visible spectrum and a high fluorescence quantum yield [33,34,35]. These synthetic molecules are important technological materials with applications in many fields such as electrochemistry [36,37,38], chemical sensors [39,40], nanomedicine [41,42,43], liquid crystals [44], anti-cancer agents [45], optical data storage [46,47], and catalysis [48,49].

Using phthalocyanines (Pcs) for Co^2+^ detection or treatment following sample preparation can be highly effective due to several compelling reasons, especially in environmental, analytical, or biomedical contexts. Phthalocyanines have a central cavity capable of coordinating metal ions like Co^2+^ through strong ligand–metal interactions. The nitrogen donor atoms within the macrocyclic structure selectively bind to transition metals, while peripheral substituents can be strategically modified to enhance selectivity and sensitivity toward Co^2+^. The primary rationale for selecting 9-(methylaminomethyl)anthracene groups in this study lies in the fact that 9-(methylaminomethyl)anthracene (MAMA) has been utilized as a model drug in investigating the impact of enzymatic degradation on the release behaviors of MAMA, as determined through UV-Vis spectroscopy [50]. The second reason is that these groups showed excellent catalytic performance in the oxidation reaction of Co^2+^ and Cu^2+^ phthalocyanine compounds to 2,3,6-trimethylphenol compound which we have previously performed [51]. This study aimed to develop and validate a novel dispersive liquid–liquid microextraction (DLLME) method integrated with UV-Vis spectrophotometry for the quantification of Co^2+^ ions. Initially, a new phthalocyanine compound, capable of forming a selective complex with Co^2+^, was synthesized and fully characterized. The optimized DLLME procedure was subsequently validated in terms of its analytical performance.

## 2. Results and Discussion

### 2.1. Characterisation of MAMA-Ni(II)Pc 2

**Figure 1:**
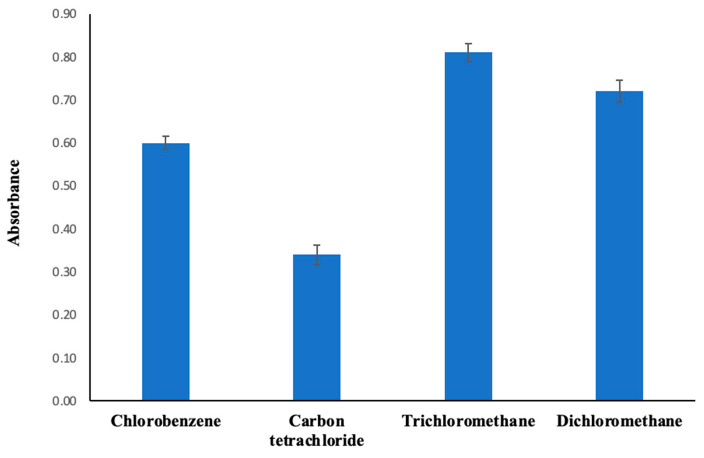
Change in absorbance depending on the extraction solvent. Extraction conditions: sample volume, 10.00 mL; dispersive solvent, 1.50 mL methanol; 350 µL of MAMA-Ni(II)Pc **2** (0.08 mmol/L, acetone); pH, 5.5; centrifugation conditions: 4000 rpm, 5 min. Error bars represent the standard deviation for n = 3.

As a result of the cyclotetramerization reaction of 4-[9-anthryl(methyl)amino]phthalonitrile 1 in the presence of DBU and a metal salt NiCl2 in 1-pentanol with good yields, 4-[9-anthryl(methyl)amino]oxy substituted Ni(II) phthalocyanine [MAMA-Ni(II)Pc **2**] was obtained (Figure 1). MAMA-Ni(II)Pc **2** was eventually characterized using spectroscopic data (UV-Vis, FT-IR, ^1^H-NMR, ^13^C-NMR, and MALDI-TOF techniques). The results of the spectral investigation of the novel product agreed with the proposed structures. MAMA-Ni(II)Pc **2** was purified through column chromatography on basic alumina, utilizing [(CHCl_3_:CH_3_OH, 94:6)] solvent mixtures as eluents. The IR spectrum revealed a sharp, characteristic peak at 2229 cm^−1^, attributed to the C≡N stretching vibration of phthalonitrile. Here, by comparing the IR spectrum absorption bands between 4-[9-anthryl(methyl)amino]phthalonitrile 1 and Ni(II) phthalocyanines, the disappearance of the characteristic C≡N stretching vibration peak of phthalonitrile at 2229 cm^−1^ in all phthalocyanines suggests the formation of phthalocyanine (Supplementary Figure S1). All metallophthalocyanine compounds display almost similar IR absorption bands. In the ^1^H NMR spectrum of MAMA-Ni(II)Pc **2**, the aromatic protons were obtained at 8.35–7.36 ppm, and aliphatic protons were obtained at 3.38 ppm. The mass spectra of MAMA-Ni(II)Pc **2**, which showed a peak at *m*/*z* = 1332.33 [M-4CH_3_]^+^, support the proposed formula for that compound (Supplementary Figure S2).

MAMA-Ni(II)Pc **2** was soluble in a wide range of organic solvents, including tetrahydrofuran (THF), dichloromethane, chloroform, DMF, DMSO, and acetone. In the CHCl_3_ solution of MAMA-Ni(II)Pc **2**, the Q bands corresponding to π-π transitions were recorded at 686 nm. The shoulders of MAMA-Ni(II)Pc **2** were observed at 605 nm. In the Q band regions of compound **2**, the longer wavelength absorptions are due to the monomeric species, and shorter wavelength absorptions are due to the aggregated species [52,53,54]. The monomeric behaviour of MPcs was proved by the dominance of the longer wavelength absorptions in CHCl_3_ at 1 × 10^−5^ mol dm^−3^ concentration. B bands arising from deeper π levels to LUMO were observed at 328 nm and 347 nm for compounds MAMA-Ni(II)Pc **2** (Supplementary Figure S3).

### 2.2. Optimization of DLLME

#### 2.2.1. Specification of Extraction Solvent Kind and Quantity

The decision as to which extraction solvent to use is an extremely important part of the DLLME process. It is essential for the extraction solvent to demonstrate a significant extraction capacity for the target analyte, and its solubility in the aqueous phase should be minimal. In addition, its density should be above the level observed for water. This will facilitate phase separation via centrifugation. It should also be compatible with the analytical instruments that will be used to determine analyte levels [55].

In this regard, chlorobenzene, carbon tetrachloride, trichloromethane, and dichloromethane were subjected to evaluation as extraction solvents. The absorbance was the highest when the trichloromethane solvent was in use (Figure 1).

**Figure 2:**
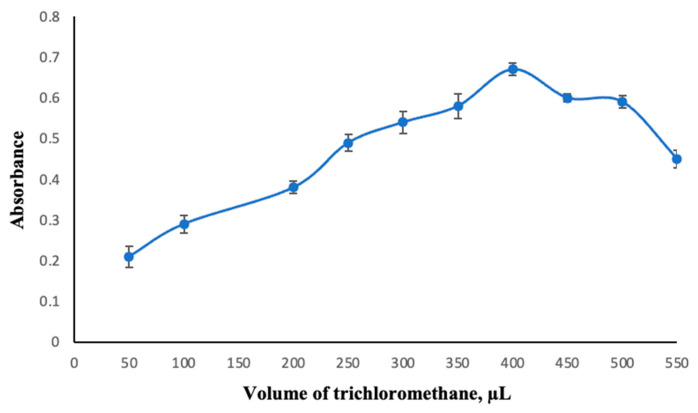
Change in absorbance depending on the extraction solvent volume. Extraction conditions: sample volume, 10.00 mL; dispersive solvent, 1.50 mL methanol; 350 µL of MAMA-Ni(II)Pc **2** (0.08 mmol/L, acetone); pH, 5.5; centrifugation conditions: 4000 rpm, 5 min. Error bars represent the standard deviation for n = 3.

The efficiency of the DLLME process was evaluated using varying volumes of trichloromethane (50 to 550 µL), while keeping the dispersive solvent volume and other conditions constant. Based on the results obtained, 400 µL of trichloromethane was identified as the optimal volume (Figure 2).

#### 2.2.2. Specification of Dispersive Solvent Kind and Quantity

**Figure 3:**
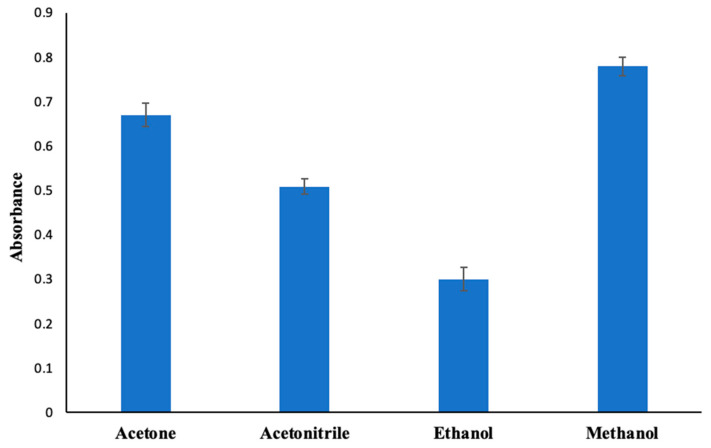
Change in absorbance depending on the dispersive solvent. Extraction conditions: sample volume, 10.00 mL; extraction solvent, 400 μL of trichloromethane; 350 µL of MAMA-Ni(II)Pc **2** (0.08 mmol/L, acetone); pH, 5.5; centrifugation conditions: 4000 rpm, 5 min. Error bars represent the standard deviation for n = 3.

The principal consideration in the decision-making process for selecting the dispersive solvent is its miscibility with both the extraction solvent and the water-based phase [56]. Acetone, acetonitrile, ethanol, and methanol, commonly used dispersive solvents, were tested for our proposed DLLME method. Aligned with the results obtained (Figure 3), methanol was selected as the dispersive solvent and utilized in the subsequent experiments.

**Figure 4:**
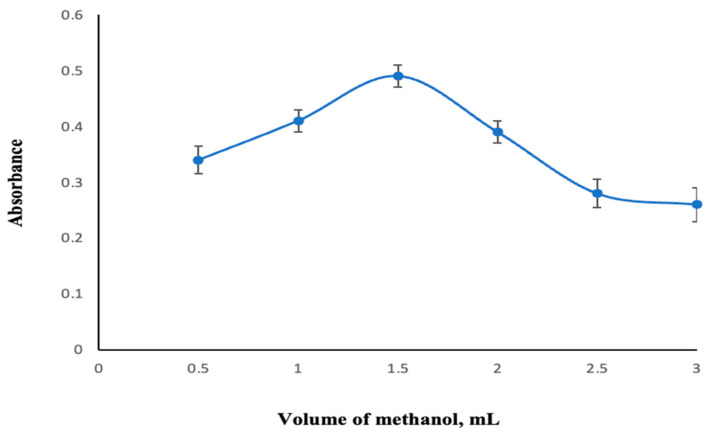
Change in absorbance depending on the dispersive solvent volume. Extraction conditions: sample volume, 10.00 mL; extraction solvent, 400 μL of trichloromethane; 350 µL of MAMA-Ni(II)Pc **2** (0.08 mmol/L, acetone); pH, 5.5; centrifugation conditions: 4000 rpm, 5 min. Error bars represent the standard deviation for n = 3.

To investigate the effect of the dispersing solvent volume, experiments were carried out using methanol volumes varying from 0.50 to 3.00 mL. When the results were examined, 1.50 mL of methanol was determined as the optimum volume in terms of extraction efficiency (Figure 4).

#### 2.2.3. Effect of pH

**Figure 5:**
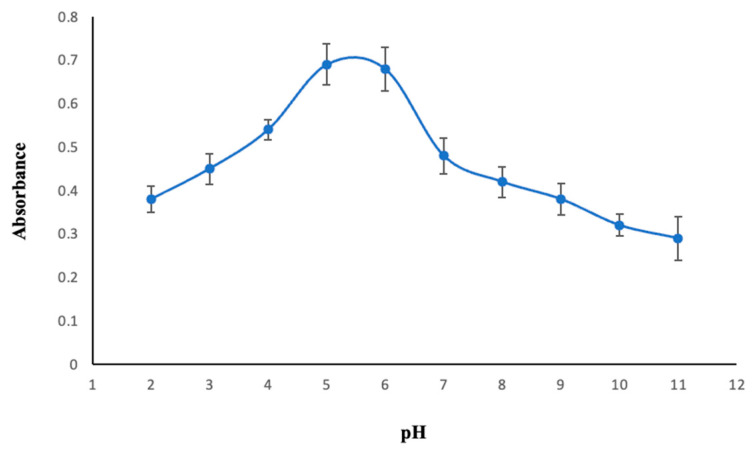
The effect of pH on the extraction of Co^2+^. Extraction conditions: sample volume, 10.00 mL; extraction solvent, 400 μL of trichloromethane; dispersive solvent, 1.50 mL methanol; 350 µL of MAMA-Ni(II)Pc **2** (0.08 mmol/L, acetone); centrifugation conditions: 4000 rpm, 5 min. Error bars represent the standard deviation for n = 3.

The pH of the aqueous phase has a substantial action in the DLLME process, as this parameter directly influences the formation and extraction of the Co^2+^-MAMA Ni(II)Pc **2** complex with sufficient hydrophobicity [57]. The extraction efficiencies at various pH levels (2.0–11.0) were examined to determine the optimum pH for the DLLME process. At low pH, the nitrogen atoms located on the peripheral substituents of MAMA-Ni(II)Pc **2** become protonated, thereby reducing the ligand’s coordination ability. At high pH, on the other hand, Co^2+^ ions tend to precipitate in the form of hydroxide, which also diminishes complex formation. It is evident from Figure 5 that the highest absorbance of Co^2+^-MAMA Ni(II)Pc **2** was observed between pH 5 and 6. Consequently, pH 5.5 was determined as optimal for subsequent analyses.

#### 2.2.4. Effect of Extraction Time

Extraction time refers to the interval between the injection of the dispersive/extraction solvent mixture until the commencement of centrifugation [58]. The relationship between extraction time and cobalt extraction was studied over a range of 5 to 15 min, with experimental conditions held constant. The findings from the experiments revealed that the extraction time had no significant effect on extraction efficiency. Notably, maximum absorbance was achieved at the 5th minute and remained stable through the 15th minute. This behaviour is consistent with the short extraction time produced by the vast surface area between the extractive solvent and the sample phase in the DLLME method [59,60].

#### 2.2.5. Concentration of Sensor

**Figure 6:**
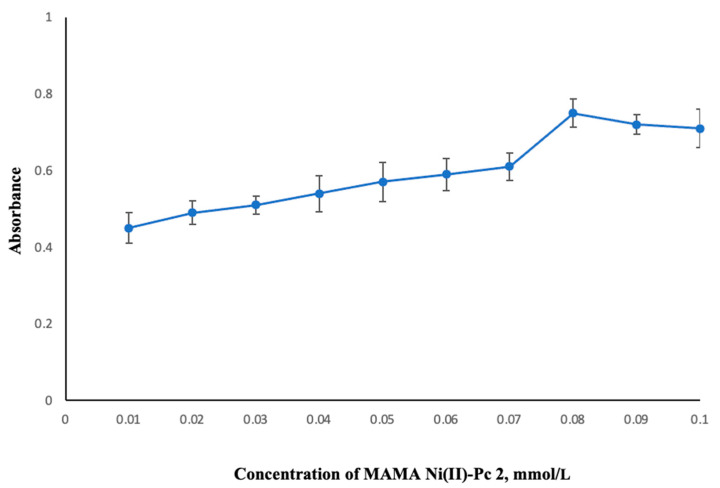
The effect of MAMA Ni(II)Pc **2** concentration on the extraction of Co^2+^. Extraction conditions: sample volume, 10.00 mL; extraction solvent, 400 μL of trichloromethane; dispersive solvent, 1.50 mL methanol; pH, 5.5; centrifugation conditions: 4000 rpm, 5 min. Error bars represent the standard deviation for n = 3.

The proposed DLLME approach is predicated on the principle of transferring the Co^2+^-MAMA Ni(II)Pc **2** complex from the aqueous sample phase to the organic phase. In this respect, it is indispensable to optimize the concentration of the sensor compound used to form the Co^2+^-MAMA Ni(II)Pc **2** complex. The relationship between MAMA Ni(II)Pc **2** concentration from 0.01 to 0.10 mmol/L and extraction efficiency was evaluated by keeping the other parameters constant. The highest absorbance was observed at a sensor concentration of 0.08 mmol/L (Figure 6).

#### 2.2.6. Centrifuge Parameters

Centrifugation is necessary to obtain a clear phase separation. To maximize efficiency at this stage, centrifuge time and speed were studied between 1.0 and 10.0 min and 1000 and 6000 rpm, respectively. Based on the experimental results, 4000 rpm and 5 min were appraised as the optimal parameters for centrifugation.

### 2.3. Interferences

The influence of potentially interfering cations on cobalt quantification with the DLLME method using MAMA Ni(II)Pc **2** was investigated. The absorbance values of mixtures prepared through the addition of foreign ions to solutions containing 0.08 mM sensor and 20 µg/L Co^2+^ were evaluated against those of cation-free solutions. The highest interfering metal concentration that did not cause less than a 5% change in absorbance was considered as the limit of tolerance and is summarized in Table 1.

### 2.4. Analytical Figures of Merit

To assess the analytical performance of the proposed DLLME method under optimal conditions, several experiments were carried out. A series of experiments, encompassing the linear range, correlation coefficient (R^2^), limit of detection (LOD), limit of quantification (LOQ), enrichment factor (EF), and precision, were executed under optimized conditions, and the outcomes are presented in Table 2.

**Figure 7:**
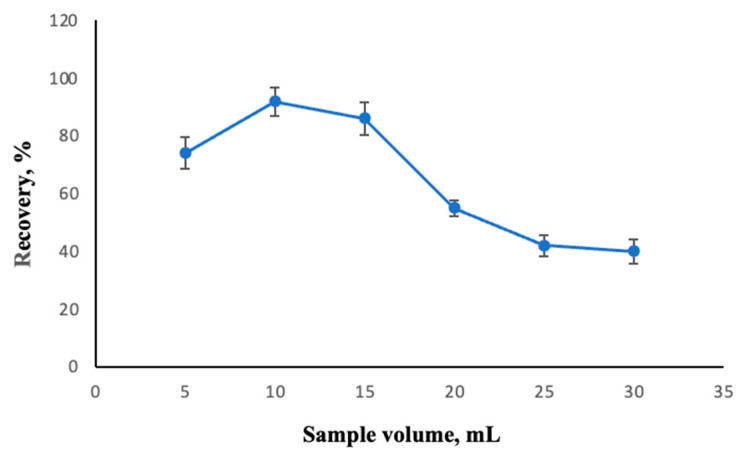
The effect of sample volume on the recovery of Co^2+^. Extraction conditions: sample volume, 10.00 mL; extraction solvent, 400 μL of trichloromethane; dispersive solvent, 1.50 mL methanol; 350 µL of MAMA-Ni(II)Pc **2** (0.08 mmol/L, acetone); pH, 5.5; centrifugation conditions: 4000 rpm, 5 min. Error bars represent the standard deviation for n = 3.

The linearity obtained for Co^2+^ analysis was observed to be between 0.40 and 260 µg/L, with a correlation coefficient (R^2^) of 0.9978. The LOD was determined to be 0.19 µg/L, based on a signal-to-noise ratio of 3. The LOQ was also determined to be 0.46 µg/L, corresponding to a signal-to-noise ratio of 10. The enrichment factor (EF) was calculated as 40 by taking the ratio of the highest sample volume at which the analyte was quantitatively recovered, 10 mL, to the final volume after extraction, 250 µL. For starters, a series of solutions containing 0.04 mg of ligand and varying between 5 and 20 mL of sample volume were analyzed with the developed method, and the optimum sample volume was found to be 10 mL. The results are shown in Figure 7. To specify the precision of this method in two aspects as both intra-day and inter-day, the analysis of 50 µg/L Co^2+^ was carried out and the relative standard deviations (RSD) were found to be 1.7% and 2.4% (n:7), respectively.

Analyte addition/recovery studies were performed on real water sample matrices to test the accuracy of the developed method. In line with this aim, 50 µg/L of Co^2+^ was spiked into three different real water samples and analyzed using the developed method. The outcomes of analyte addition/recovery studies in water samples are presented in Table 3.

### 2.5. Comparison with Other Studies

Table 4 provides a selection of recent studies on the determination of Co^2+^ using the DLLME method. A scientific method involving the microextraction of Co^2+^ after complexation with 1-[4-[(2-hydroxynaphthalen-1-yl)methylideneamino] phenyl]ethanone (HNE), used a binary mixture of methanol and chloroform as the dispersive and extraction solvents in a volume ratio of 900:400 µL. The centrifugation criteria yielding the best performance were found to be 5000 rpm for 2 min. The absorbance of the organic phase diluted with 1 mL of methanol was measured at 324 nm in a quartz microcell with a long optical path (50 mm) and a thickness of 9 mm. The method exhibited a linear range of 0.45–10.0 µg/L, an LOD of 0.08 µg/L, an LOQ µg/L of 3, and an RSD of 1.6% [61]. Another scientific publication focused on the simultaneous extraction of V, Ni, Cu, Zn, Se, Mo, Cd, Pb, and Co cations from seawater. As chelating agents for all metal cations, the study utilized ammonium pyrrolidinedithiocarbamate and diethyldithiocarbamic acid diethylammonium salts. Optimal conditions included the use of 400 µL of chelating reagent, methanol (2 mL) as the dispersive solvent, chloroform (1 mL) as the extraction solvent, and an adjusted pH of 3. To release the metal ions, 0.5 mL of nitric acid was added to the residue, followed by heating. The solution was then diluted to 1 mL with deionized water and analyzed using ICP-MS. For the Co^2+^ ion, the method demonstrated a linear range between 0.1 and 100 µg/L, with an LOD of 0.02 µg/L, an LOQ of 0.07 µg/L, and an RSD of 2.7% [62]. A separate article applying DLLME for Cu^2+^ and Co^2+^ extraction reported optimal conditions of 0.05 mg dithizone (chelating agent), pH 6, 250 µL chloroform (extraction solvent), and 1000 µL acetone (dispersive solvent). Following evaporation of the organic phase in a water bath, the residue was dissolved in 100 μL of concentrated nitric acid. The solution was subsequently diluted to 250 μL with deionized water and analyzed for cation content using FAAS. The analytical performance of the method for Co^2+^ included a linear range of 0–6 mg/L, an LOD of 2.48 µg/L, an LOQ of 9.01 µg/L, and an RSD of 1% [63]. In another study, cobalt was complexed with SAN and extracted using a DLLME procedure involving 60 μL of carbon tetrachloride (CTC) and 1000 μL of ethanol as the extraction and dispersive solvents, respectively. The method was validated with a linear range of 4.00–160 µg/L, an LOD of 1.04 µg/L, an LOQ of 3.47 µg/L, and an RSD value varying from 2.4% to 11.8% [64].

As demonstrated in Table 4, our developed extraction protocol exhibits reliable and comparable performance to the methods reported in the recent literature, in which DLLME-based approaches involve the complexation of Co^2+^ ions with various chelating reagents prior to their extraction from the sample matrix. Al-Saidi et al. [61] utilized DLLME combined with long-path microcells for cobalt determination, but their method showed a relatively narrower linear range and higher LOD. Sajid et al. [62] reported a DLLME-ICP-MS approach for multi-element detection in seawater. While the method offers high sensitivity and selectivity, it necessitates the use of expensive and sophisticated instrumentation, limiting its routine applicability. Divrikli et al. [63] presented a green DLLME-FAAS method for Co and Cu but with a relatively lower enhancement factor and narrower dynamic range. Mehdi et al. [64] achieved effective pre-concentration of Co^2+^ with DLLME prior to FAAS, yet their reported LOD and RSD values were slightly higher than those obtained in this study.

## 3. Materials and Methods

### 3.1. Reagents and Instrumentation

All chemicals and solvents were purchased from Merck (Darmstadt, Germany) and Sigma-Aldrich (Taufkirchen, Germany) and were of analytical grade. The stock standard solutions of cations were prepared at a concentration of 1000 mgL^−1^ from nitrate salts in methanol. The sensor compound solution was prepared by dissolving an appropriate amount of reagent in 25 mL of acetone and kept at 4 °C for a week. 4-[9-anthryl(methyl)amino] phthalonitrile was prepared according to the literature procedure [51]. 4-nitro phthalonitrile and 9-(methylaminomethyl)anthracene (MAMA) were purchased from Sigma-Aldrich and used without further purification and chemical treatment.

All details for instrumentation are provided in the Supplementary File.

### 3.2. Synthesis of MAMA-Ni(II)Pc **2**

**Figure 8:**
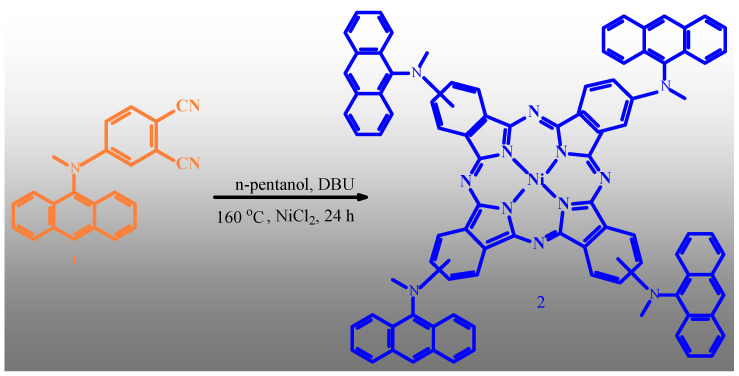
Synthesis of MAMA-Ni(II)Pc **2** (n-pentanol, DBU, NiCl2, 160 °C, 24 h).

A mixture of 4-[9-anthrylmethyl)amino]phthalonitrile (1) (1.6 g, 4.78 mmol), anhydrous nickel(II) chloride (NiCl_2_) (154.94 mg, 1.196 mmol), and five drops of DBU was subjected to heating at 160 °C in dry n-pentanol (6 mL) under sealed-tube conditions and stirred for 24 h (Figure 8). After completion of the reaction, the green precipitate was obtained through the addition of ethanol (40 mL) and collected via filtration. For 2 h, the green solid product was refluxed with ethanol (60 mL), filtered off again, and washed with hot ethanol, distilled water, and diethyl ether. Following vacuum drying, the product was subjected to purification via column chromatography over basic alumina, employing chloroform–methanol (94:6, *v*/*v*) as the eluent. Yield: 800 mg (45%). Mp > 300 °C. Anal. calc. for C_92_H_60_N_12_Ni FT-IR ν_max_/cm^−1^ (KBr pellet): 3078 (Ar-H), 2938–2893 (Aliphatic. C-H), 1600, 1581, 1452, 1438, 1426, 1372, 1353, 1262, 1200, 1150, 10,877, 1039, 964, 946, 912, 839, 803. ^1^H-NMR (CDCl_3_), (δ: ppm): 7.36–7.42 (m, 8H, ArH), 7.55–7.61 (m, 16H, ArH), 7.80–7.99 (m, 8H, ArH), 8.05–8.15 (m, 8H, ArH), 8.22–8.35 (m,8H, ArH), 3.38 (s, 12H, H_3_C-N). UV-Vis (CHCI_3_): λ_max_, nm (log ε): 686 (4.96), 625 (4.86), 605 (4.38), 347 (4.88), 328 (5.02). MALDI-TOF-MS, (*m*/*z*): calculated: 1392.25; found: 1332.33 [M-4CH_3_]^+^.

### 3.3. DLLME Procedure

**Figure 9:**
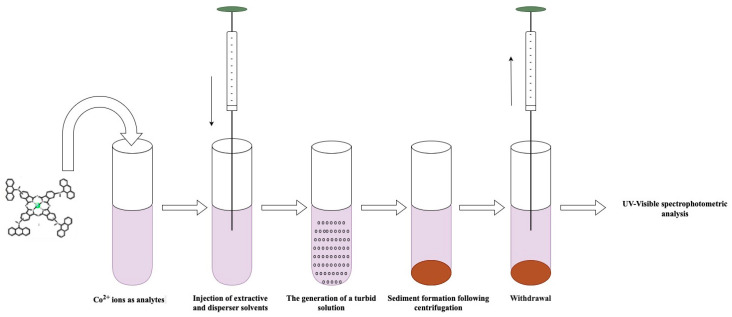
Schematic illustration of the proposed DLLME procedure.

First, 350 µL of MAMA-Ni(II)Pc **2** (0.08 mmol/L, acetone) was added as a chelating agent to 10 mL of aqueous sample solution containing Co^2+^ in a 15 mL conical test tube. After adjustment of the pH of the environment to 5.5, Co^2+^ cations reacted with MAMA-Ni(II)Pc **2** to form a hydrophobic complex. The mixture containing 1.50 mL of methanol as the dispersive solvent and 400 µL of trichloromethane as the extraction solvent was rapidly injected into the sample solution using a 2.0 mL syringe. The solution clouded quickly, and the Co^2+^ ion complex was moved into the trichloromethane droplets within a few seconds. Then, the tube was centrifuged at 4000 rpm for 5 min. and the aqueous supernatant was removed with a syringe. The volume of the extraction phase was diluted to 250 µL with trichloromethane. The resulting sample solution was then transferred to a 250 mL quartz cell for absorbance measurement at 350 nm (Figure 9).

## 4. Conclusions

This research presents the development of a rapid, simple, cost-effective, efficient, robust, and safe extraction technique integrated with dispersive liquid–liquid microextraction (DLLME) for the trace determination of Co^2+^ ions in aqueous samples. The proposed method relies on the extraction of Co^2+^ ions into the organic phase following their complexation with MAMA-Ni(II)Pc **2**, a novel phthalocyanine derivative. Extraction is facilitated by a solvent system composed of 1.50 mL methanol as the dispersive solvent and 400 µL trichloromethane as the extraction solvent. The literature review reveals that studies involving the use of phthalocyanine compounds as complexing agents in DLLME are quite limited. One of the few studies using phthalocyanine ligands as complexing reagents was conducted by Çağlar et al. [65], who proposed the use of a Fe^2+^ phthalocyanine for the determination of mercury (Hg) in water samples using the DLLME method. This indicates that the potential of phthalocyanine derivatives in the DLLME method, particularly for the preconcentration and determination of metal ions, has not been sufficiently explored, highlighting a significant gap in the literature. Therefore, evaluating such compounds in DLLME-based analyses could contribute to the development of novel and original applications in the fields of environmental and analytical chemistry. The proposed method is represented as an environmentally friendly analytical protocol that complies with the principles of green chemistry by minimizing the consumption of organic solvents and the generation of waste. Additionally, it offers a wide linear range, low LOD and LOQ, a high enrichment factor, and practical applicability using cost-effective instrumentation such as UV-Vis spectrophotometry.

## Figures and Tables

**Table 1:** Tolerance limits of co-existing ions for the determination of Co^2+^ (20 µg/L).

Foreign Ion Added	Tolerance Limit (µg/L)
Na^+^	650
K^+^	800
Mg^2+^	850
Ca^2+^	400
Ba^2+^	300
Mn^2+^	752
Fe^3+^	100
Ni^2+^	50
Cu^2+^	150
Zn^2+^	200
Cd^2+^	300
Pb^2+^	300
Cr^2+^	150

**Table 2:** Evaluation of analytical figures of merit for cobalt determination.

Parameters	
Linear range, µg/L	0.40–260
Correlation coefficient (R^2^)	0.9978
LOD, µg/L	0.19
LOQ, µg/L	0.46
Enhancement factor,	40
RSD, % (n = 7)	1.7 ^a^ and 2.4 ^b^

^a^ Intra-day precision; ^b^ inter-day precision.

**Table 3:** Studies on analyte addition/recovery in drinking water sample matrices for accuracy testing.

Sample	Co^2+^ Amount µg/L		
	Added	Finded	Recovery % ± s.d. ^a^
Tap water 1	25 50.00	24.18 52.31	96.72 ± 4.1 104.62 ± 3.9
Tap water 2	25 50.00	23.89 52.45	95.56 ± 2.7 104.9 ± 5.2
Tap water 3	25 50.00	24.22 48.24	96.88 ± 2.5 96.48 ± 3.2

^a^ s.d., standard deviation.

**Table 4:** Comparative evaluation of the developed DLLME method with other reported DLLME techniques for Co^2+^ quantification.

Extraction Method	Linear Range (µg/L)	LOD (µg/L)	LOQ (µg/L)	RSD (%)	Reference
DLLME	0.45–10.0	0.08	0.264	1.6	[61]
DLLME ^a^	0.1–100	0.02 ^b^	0.07	2.7	[62]
DLLME ^a^	0–6 ^b^	2.48 ^b^	9.01	1	[63]
DLLME	4.00–160	1.04	3.47	2.4–11.8	[64]
DLLME	0.40–260	0.19	0.46	1.7 ^c^ and 2.4 ^d^	Present study

^a^ The Co^2+^ parameters extracted from the simultaneous detection of multiple ions. ^b^ The linear range was reported in mg/L. ^c^ Intra-day precision. ^d^ Inter-day precision.

## Data Availability

The original contributions presented in this study are included in the article and Supplementary Material.

## Supplementary Materials

The following supporting information can be downloaded at https://www.mdpi.com/article/10.3390/molecules30122548/s1. Figure S1. FT-IR spectra of MAMA-Ni(II)Pc 2. Figure S2. MALDI-TOF spectra of MAMA-Ni(II)Pc **2**. Figure S3. UV-Vis spectrum of MAMA-Ni(II)Pc **2** in CHCl_3_ at 1 × 10^−5^ mol dm^−3^ concentration. Refs. [66,67] are cited in the Supplementary Materials.

## References

[1] Thakur A., Kumar A. (2022). Recent Advances on Rapid Detection and Remediation of Environmental Pollutants Utilizing Nanomaterials-Based (Bio)Sensors. Sci. Total Environ..

[2] Cerrato A., Cannazza G., Capriotti A.L., Citti C., La Barbera G., Laganà A., Montone C.M., Piovesana S., Cavaliere C. (2020). A New Software-Assisted Analytical Workflow Based on High-Resolution Mass Spectrometry for the Systematic Study of Phenolic Compounds in Complex Matrices. Talanta.

[3] Zhou Y., Wei X., Huang L., Wang H. (2023). Worldwide Research on Extraction and Recovery of Cobalt through Bibliometric Analysis: A Review. Environ. Sci. Pollut. Res..

[4] Gray J.E., Eppinger R.G. (2012). Distribution of Cu, Co, As, and Fe in Mine Waste, Sediment, Soil, and Water in and around Mineral Deposits and Mines of the Idaho Cobalt Belt, USA. Appl. Geochem..

[5] Czarnek K., Terpilowska S., Siwicki A.K. (2015). Selected Aspects of the Action of Cobalt Ions in the Human Body. Cent. Eur. J. Immunol..

[6] Simonsen L.O., Harbak H., Bennekou P. (2012). Cobalt Metabolism and Toxicology—A Brief Update. Sci. Total Environ..

[7] Santos A.J.M., Khemiri S., Simões S., Prista C., Sousa I., Raymundo A. (2023). The Importance, Prevalence and Determination of Vitamins B6 and B12 in Food Matrices: A Review. Food Chem..

[8] Bhattacharya P.T., Misra S.R., Hussain M. (2016). Nutritional Aspects of Essential Trace Elements in Oral Health and Disease: An Extensive Review. Scientifica.

[9] Behbahani M., Zarezade V., Veisi A., Omidi F., Bagheri S. (2019). Modification of Magnetized MCM-41 by Pyridine Groups for Ultrasonic-Assisted Dispersive Micro-Solid-Phase Extraction of Nickel Ions. Int. J. Environ. Sci. Technol..

[10] Rohani Moghadam M., Poorakbarian Jahromi S.M., Darehkordi A. (2016). Simultaneous Spectrophotometric Determination of Copper, Cobalt, Nickel and Iron in Foodstuffs and Vegetables with a New Bis Thiosemicarbazone Ligand Using Chemometric Approaches. Food Chem..

[11] Tekin Z., Erarpat S., Şahin A., Selali Chormey D., Bakırdere S. (2019). Determination of Vitamin B12 and Cobalt in Egg Yolk Using Vortex Assisted Switchable Solvent Based Liquid Phase Microextraction Prior to Slotted Quartz Tube Flame Atomic Absorption Spectrometry. Food Chem..

[12] Shirani M., Habibollahi S., Akbari A. (2019). Centrifuge-Less Deep Eutectic Solvent Based Magnetic Nanofluid-Linked Air-Agitated Liquid–Liquid Microextraction Coupled with Electrothermal Atomic Absorption Spectrometry for Simultaneous Determination of Cadmium, Lead, Copper, and Arsenic in Food Samples and Non-Alcoholic Beverages. Food Chem..

[13] Gouda A.A., El Sheikh R., El Sayed H.M., Khedr A.M., Al Ezz S.A., Gamil W., Hamdy M. (2022). Ultrasound-Assisted Dispersive Microsolid-Phase Extraction for Preconcentration of Trace Cobalt and Nickel in Environmental Samples Prior to Their Determination by Flame Atomic Absorption Spectrometry. J. Appl. Spectrosc..

[14] Jamila N., Khan N., Hwang I.M., Saba M., Khan F., Amin F., Khan S.N., Atlas A., Javed F., Minhaz A. (2020). Characterization of Natural Gums via Elemental and Chemometric Analyses, Synthesis of Silver Nanoparticles, and Biological and Catalytic Applications. Int. J. Biol. Macromol..

[15] Min Hwang I., Yang J.-S., Hyun Kim S., Jamila N., Khan N., Su Kim K., Seo H.-Y., Hwang M. (2016). Elemental Analysis of Sea, Rock, and Bamboo Salts by Inductively Coupled Plasma-Optical Emission and Mass Spectrometry. Anal. Lett..

[16] Smirnova S.V., Ilin D.V., Pletnev I.V. (2021). Extraction and ICP-OES Determination of Heavy Metals Using Tetrabutylammonium Bromide Aqueous Biphasic System and Oleophilic Collector. Talanta.

[17] Sadia M., Rasul Jan M., Shah J., Greenway G.M. (2013). Simultaneous Preconcentration and Determination of Nickel and Cobalt Using Functionalised Mesoporous Silica Spheres by ICP-OES. Int. J. Environ. Anal. Chem..

[18] Feist B., Mikula B. (2014). Preconcentration of Heavy Metals on Activated Carbon and Their Determination in Fruits by Inductively Coupled Plasma Optical Emission Spectrometry. Food Chem..

[19] Shirani M., Salari F., Habibollahi S., Akbari A. (2020). Needle Hub In-Syringe Solid Phase Extraction Based a Novel Functionalized Biopolyamide for Simultaneous Green Separation/Preconcentration and Determination of Cobalt, Nickel, and Chromium (III) in Food and Environmental Samples with Micro Sampling Flame Atomic Absorption Spectrometry. Microchem. J..

[20] Arain M.B., Yilmaz E., Soylak M. (2016). Deep Eutectic Solvent Based Ultrasonic Assisted Liquid Phase Microextraction for the FAAS Determination of Cobalt. J. Mol. Liq..

[21] Loṕez-García I., Vicente-Martińez Y., Hernańdez-Córdoba M. (2013). Determination of Lead and Cadmium Using an Ionic Liquid and Dispersive Liquid-Liquid Microextraction Followed by Electrothermal Atomic Absorption Spectrometry. Talanta.

[22] Malik M., Chan K.H., Azimi G. (2021). Quantification of Nickel, Cobalt, and Manganese Concentration Using Ultraviolet-Visible Spectroscopy. RSC Adv..

[23] Zhou F., Li C., Zhu H., Li Y. (2019). Determination of Trace Ions of Cobalt and Copper by UV–Vis Spectrometry in Purification Process of Zinc Hydrometallurgy. Optik.

[24] Ünaldı M., Yıldız B., Durukan İ. (2024). Green Surfactant Assisted-Solidified Floating Organic Drop Micro-Extraction for the Preconcentration of Trace Cobalt and Determination by Flame Atomic Absorption Spectrometry. Anal. Lett..

[25] Ghoochani Moghadam A., Rajabi M., Hemmati M., Asghari A. (2017). Development of Effervescence-Assisted Liquid Phase Microextraction Based on Fatty Acid for Determination of Silver and Cobalt Ions Using Micro-Sampling Flame Atomic Absorption Spectrometry. J. Mol. Liq..

[26] Yazıcı E., Fırat M., Selali Chormey D., Gülhan Bakırdere E., Bakırdere S. (2020). An Accurate Determination Method for Cobalt in Sage Tea and Cobalamin: Slotted Quartz Tube-Flame Atomic Absorption Spectrometry after Preconcentration with Switchable Liquid-Liquid Microextraction Using a Schiff Base. Food Chem..

[27] Mandal S., Lahiri S. (2022). A Review on Extraction, Preconcentration and Speciation of Metal Ions by Sustainable Cloud Point Extraction. Microchem. J..

[28] Rezaee M., Assadi Y., Milani Hosseini M.R., Aghaee E., Ahmadi F., Berijani S. (2006). Determination of Organic Compounds in Water Using Dispersive Liquid-Liquid Microextraction. J. Chromatogr. A.

[29] Al-Saidi H.M., Emara A.A.A. (2014). The Recent Developments in Dispersive Liquid-Liquid Microextraction for Preconcentration and Determination of Inorganic Analytes. J. Saudi Chem. Soc..

[30] Faraji H. (2024). Advancements in Overcoming Challenges in Dispersive Liquid-Liquid Microextraction: An Overview of Advanced Strategies. TrAC—Trends Anal. Chem..

[31] Kamal El-Deen A., Elmansi H., Belal F., Magdy G. (2023). Recent Advances in Dispersion Strategies for Dispersive Liquid–Liquid Microextraction from Green Chemistry Perspectives. Microchem. J..

[32] Oflu S., Zaman B.T., Kılınç Y., Bakırdere S., Turak F. (2024). Determination of Trace Amounts of Metobromuron Herbicide Residues in Fruits by QuEChERS and DLLME Methods. J. Food Compos. Anal..

[33] Senge M.O., Sergeeva N.N., Hale K.J. (2021). Classic Highlights in Porphyrin and Porphyrinoid Total Synthesis and Biosynthesis. Chem. Soc. Rev..

[34] Gounden D., Nombona N., van Zyl W.E. (2020). Recent Advances in Phthalocyanines for Chemical Sensor, Non-Linear Optics (NLO) and Energy Storage Applications. Coord. Chem. Rev..

[35] Lu H., Kobayashi N. (2016). Optically Active Porphyrin and Phthalocyanine Systems. Chem. Rev..

[36] Souza F. (2002). Recent Advances in the Electrochemistry of Porphyrins and Phthalocyanines. J. Porphyr. Phthalocyanines.

[37] Beduoğlu A., Budak Ö., Sevim A.M., Koca A., Bayır Z.A. (2021). Double-Decker Lutetium Phthalocyanine Functionalized with 4-Phenylthiazol-2-Thiol Moieties: Synthesis, Characterization, Electrochemistry, Spectroelectrochemistry and Electrochromism. Polyhedron.

[38] Rawling T., McDonagh A.M., Colbran S.B. (2008). Synthesis, Electrochemistry and Spectroscopic Properties of Ruthenium Phthalocyanine and Naphthalocyanine Complexes with Triphenylarsine Ligands. Inorganica Chim. Acta.

[39] Liu X., Qi C., Bing T., Cheng X., Shangguan D. (2009). Highly Selective Phthalocyanine-Thymine Conjugate Sensor for Hg 2+ Based on Target Induced Aggregation. Anal. Chem..

[40] Claessens C.G., Hahn U., Torres T. (2008). Phthalocyanines: From Outstanding Electronic Properties to Emerging Applications. Chem. Rec..

[41] Lo P.C., Rodríguez-Morgade M.S., Pandey R.K., Ng D.K.P., Torres T., Dumoulin F. (2020). The Unique Features and Promises of Phthalocyanines as Advanced Photosensitisers for Photodynamic Therapy of Cancer. Chem. Soc. Rev..

[42] Zheng B.D., He Q.X., Li X., Yoon J., Huang J.D. (2021). Phthalocyanines as Contrast Agents for Photothermal Therapy. Coord. Chem. Rev..

[43] Galstyan A. (2021). Turning Photons into Drugs: Phthalocyanine-Based Photosensitizers as Efficient Photoantimicrobials. Chem.-Eur. J..

[44] Reheman A., Hu S., Cao L., Xie D., Yan G., Wang J. (2021). Liquid-Crystalline Behaviour and Electrorheological Effect of Phthalocyanine-Based Ionic Liquid Crystals. Liq. Cryst..

[45] Yenilmez H.Y., Farajzadeh N., Tollu G., Kuşçulu N.G., Bahar D., Özdemir S., Bayır Z.A. (2023). Silicon Phthalocyanines as Anti-Infectious, Antioxidant, and Anticancer Agents. Chem. Sel..

[46] Li B., Wei P., de Leon A., Frey T., Pentzer E. (2017). Polymer Composites with Photo-Responsive Phthalocyanine for Patterning in Color and Fluorescence. Eur. Polym. J..

[47] Seto J., Tamura S.I., Asai N., Kishii N., Kijima Y., Matsuzawa N. (1996). Macrocyclic Functional Dyes: Applications to Optical Disk Media, Photochemical Hole Burning and Nonlinear Optics. Pure Appl. Chem..

[48] Ravikanth M., Achim C., Tyhonas J.S., Münck E., Lindsey J.S. (1997). Investigation of Phthalocyanine Catalysts for the Aerobic Synthesis of Meso-Substituted Porphyrins. J. Porphyr. Phthalocyanines.

[49] Katsurayama Y., Ikabata Y., Maeda H., Segi M., Nakai H., Furuyama T. (2022). Direct Near Infrared Light–Activatable Phthalocyanine Catalysts. Chem.-Eur. J..

[50] Spinelli F., D’Agostino S., Taddei P., Jones C.D., Steed J.W., Grepioni F. (2018). Activating [4 + 4] Photoreactivity in the Solid-State: Via Complexation: From 9-(Methylaminomethyl)Anthracene to Its Silver(i) Complexes. Dalton Trans..

[51] Saka E.T. (2018). Preparation, Characterization of New Co(II) and Cu(II) Phthalocyanines and Their Catalytic Performances in Aerobic Oxidation of Substituted Phenols. J. Incl. Phenom. Macrocycl. Chem..

[52] Saglam Ertunga N., Saka E.T., Taskin-Tok T., Inan Bektas K., Yildirim Akatin M. (2024). Synthesis, Characterization, DNA Interaction, Molecular Docking, and α-Amylase and α-Glucosidase Inhibition Studies of a Water Soluble Zn(Ii) Phthalocyanine. Dalton Trans..

[53] Saka E.T., Senocak A., Akkol C. (2024). Synthesis of Phthalocyanine/C3N4 Structures and Investigation of Photocatalytic Activities in the Oxidation Reaction of 4-Nitrophenol. J. Coord. Chem..

[54] Avlar A., Muzaffarzade Y., Tutal B., Bekircan O., Saka E.T. (2025). Synthesis of 1,2,4-Triazole Substituted Co(II) and Cu(II) Phthalocyanine Compounds and Investigation of Their Photocatalytic Activities in the Photochemical Degradation Reaction of 4-Nitrophenol. J. Mol. Struct..

[55] Quigley A., Cummins W., Connolly D. (2016). Dispersive Liquid-Liquid Microextraction in the Analysis of Milk and Dairy Products: A Review. J. Chem..

[56] Abedi A.-S., Mohammadi A., Azadniya E., Mohammad Mortazavian A., Khaksar R. (2014). Simultaneous Determination of Sorbic and Benzoic Acids in Milk Products Using an Optimised Microextraction Technique Followed by Gas Chromatography. Food Addit. Contam. Part A.

[57] Alothman Z.A., Habila M., Yilmaz E., Soylak M. (2013). A Dispersive Liquid-Liquid Microextraction Methodology for Copper(II) in Environmental Samples Prior to Determination Using Microsample Injection Flame Atomic Absorption Spectrometry. J. AOAC Int..

[58] Berijani S., Assadi Y., Anbia M., Milani Hosseini M.R., Aghaee E. (2006). Dispersive Liquid-Liquid Microextraction Combined with Gas Chromatography-Flame Photometric Detection. Very Simple, Rapid and Sensitive Method for the Determination of Organophosphorus Pesticides in Water. J. Chromatogr. A.

[59] Liang P., Sang H. (2008). Determination of Trace Lead in Biological and Water Samples with Dispersive Liquid-Liquid Microextraction Preconcentration. Anal. Biochem..

[60] Liang P., Peng L., Yan P. (2009). Speciation of As(III) and As(V) in Water Samples by Dispersive Liquid-Liquid Microextraction Separation and Determination by Graphite Furnace Atomic Absorption Spectrometry. Microchim. Acta.

[61] Al-Saidi H.M., Alharthi S.S. (2021). Efficiency Enhancement of the Spectrophotometric Estimation of Cobalt in Waters and Pharmaceutical Preparations Using Dispersive Liquid–Liquid Microextraction and Microcells with Long Optical Paths. Spectrochim. Acta Part A Mol. Biomol. Spectrosc..

[62] Sajid M., Asif M., Ihsanullah I. (2021). Dispersive Liquid–Liquid Microextraction of Multi-Elements in Seawater Followed by Inductively Coupled Plasma-Mass Spectrometric Analysis and Evaluation of Its Greenness. Microchem. J..

[63] Divrikli U., Altun F., Akdoğan A., Soylak M., Elçi L. (2021). An Efficient Green Microextraction Method of Co and Cu in Environmental Samples Prior to Their Flame Atomic Absorption Spectrometric Detection. Int. J. Environ. Anal. Chem..

[64] Mehdi Z.S., Alshamkhawy S.A.R.A. (2024). A Univariate Optimization Strategy for Pre-Concentration of Cobalt(II) in Various Matrixes by a DLLME before Analysis Using FAAS. Indones. J. Chem..

[65] Çağlar Y., Biyiklioglu Z. (2018). Spectrophotometric Determination of Hg(II) in Water Samples by Dispersive Liquid Liquid Microextraction with Use Ionic Liquid after Derivatization with a Water Soluble Fe(II) Phthalocyanine. J. Incl. Phenom. Macrocycl. Chem..

[66] Perrin D.D., Armarego W.L.F. (1989). Purification of Laboratory Chemicals.

[67] Young Y.G., Onyebuagu W. (1990). Synthesis and Characterization of Di-Disubstituted Phthalocyanines. J. Org. Chem..

