# Supplementary material for: Dispersive Liquid–Liquid Microextraction Method Utilizing a Novel Peripherally Tetra-Substituted Ni(II) Phthalocyanine as a Sensor Prior to UV-Visible Spectrophotometry for the Determination of Co2+
Source: Molecules. 2025 Jun 11;30(12):2548. doi: 10.3390/molecules30122548 (PMC12195804; doi:10.3390/molecules30122548)
Supplement: Supplementary file 1 [file molecules-30-02548-s001.zip › molecules-3644100-supplementary.pdf]

Supplementary Information

Dispersive Liquid–Liquid Microextraction Method Utilizing a Novel Peripherally Tetra-Substituted Ni(II) Phthalocyanine as a Sensor Prior to UV-Visible Spectrophotometry for the Determination of Co^{2+}

Yasemin Çağlar [1,*] and Ece Tuğba Saka [2]

[1] Department of Genetic and Bioengineering, Giresun University, Giresun 28200, Türkiye
[2] Department of Chemistry, Karadeniz Technical University, Trabzon 61080, Türkiye; esaka@ktu.edu.tr
* Correspondence: yasemin.caglar@giresun.edu.tr; Tel.: +90-454-310-40-16

Equipment

The absorbance measurements during DLLME were made on a Thermo Scientific Evuluation Array spectrophotometer (Thermo Fisher Scientific, Waltham, MA, USA) using a quartz micro cuvette with 250 μL internal capacity. The pH measurements were performed using a Hanna HI 2211 model pH meter (Hanna Instruments, Woonsocket, RI, USA) combined with a glass electrode. Kudos SK2210 HP model ultrasonic bath (Kudos Instruments Corp., New York, NY, USA) was used to help dissolve of the phthalocyanine compound. The IR spectra were acquired on a Perkin Elmer 1600 FT-IR spectrophotometer (PerkinElmer Inc., Waltham, MA, USA) using KBr pellets. ^{1}H-NMR and ^{13}C-NMR spectra were obtained with a Varian Mercury 400 MHz spectrometer (Varian, Inc., Palo Alto, CA, USA) in $CDCl_3$. All chemical shifts (δ) are given relative to tetramethylsilane (TMS) as an internal reference. MALDI-MS spectra of the complexes were obtained using dihydroxybenzoic acid as the matrix, with a nitrogen laser and accumulation of 50 laser shots, on a Bruker Microflex LT MALDI-TOF mass spectrometer (Bruker Daltonik GmbH, Bremen, Germany).

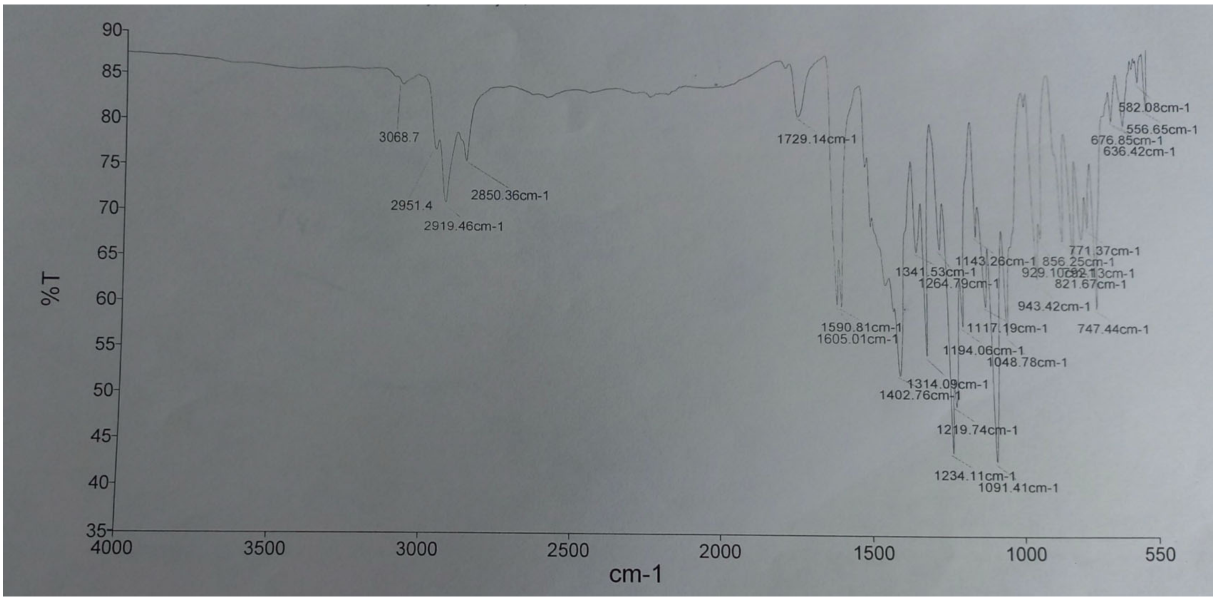

Supplementary Figure S1. FT-IR spectra of MAMA-Ni(II)Pc **2**.

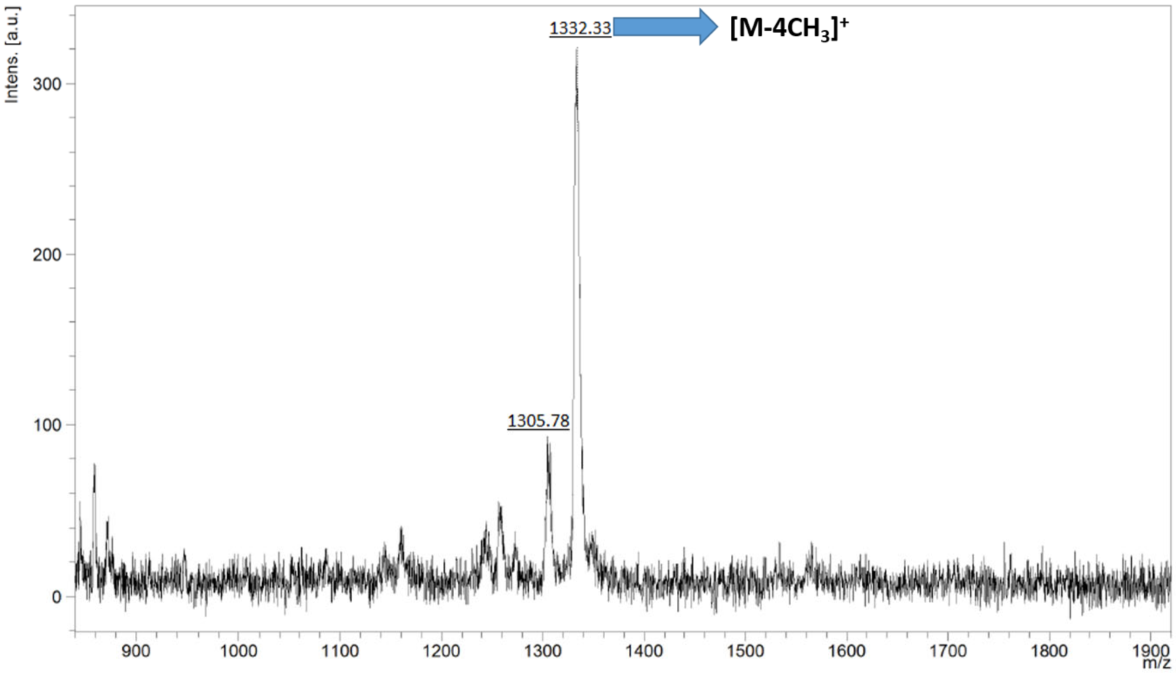

Supplementary Figure S2. MALDI-TOF spectra of MAMA-Ni(II)Pc **2**.

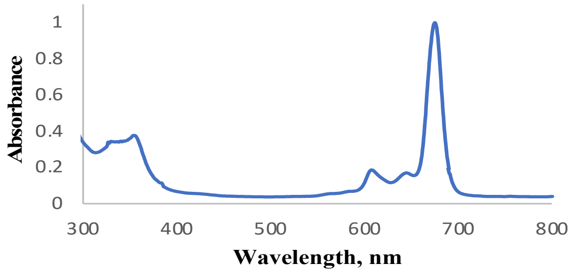

Supplementary Figure S3. UV-Vis spectrum of MAMA-Ni(II)Pc **2** in $CHCl_3$ at 1 x10^{-5} mol dm^{-3} concentration.

References

67. Perrin, D.D.; Armarego, W.L.F. *Purification of Laboratory Chemicals*, 2nd ed.; Pergamon Press: Oxford, UK, 1989.
68. Young, Y.G.; Onyebuagu, W. Synthesis and Characterization of Di-Disubstituted Phthalocyanines. *J. Org. Chem.* **1990**, *55*, 2155–2159. https://doi.org/10.1021/jo00294a032.
